# Single-cell RNA sequencing reveals compartment-specific immune and tumor cell states in a contiguous spinal metastasis

**DOI:** 10.1093/noajnl/vdag225

**Published:** 2026-08-28

**Authors:** Yassmin A Elbanna, Kenny K H Yu, Tejus A Bale, Morgan Huse, Ori Barzilai

**Affiliations:** Louis V. Gerstner, Jr., Graduate School of Biomedical Sciences, Memorial Sloan Kettering Cancer Center, New York, New York, USA; Department of Neurosurgery, Memorial Sloan Kettering Cancer Center, New York, New York, USA; Department of Pathology and Laboratory Medicine, Memorial Sloan Kettering Cancer Center, New York, New York, USA; Immunology Program, Memorial Sloan Kettering Cancer Center, New York, New York, USA; Department of Neurosurgery, Memorial Sloan Kettering Cancer Center, New York, New York, USA

**Keywords:** immune landscape, intra-tumoral heterogeneity, spinal metastasis, tumor microenvironment

## Abstract

**Background:**

Metastatic disease commonly exhibits differential treatment responses across anatomical sites, including visceral versus osseous lesions and, within the spine, osseous, epidural, and intradural compartments. Intradural spinal metastases, though rare, portend rapid neurologic deterioration and worse outcomes than epidural disease, yet the biological mechanisms underlying this differential behavior remain poorly understood.

**Methods:**

We report a 61-year-old woman with metastatic ER+/PR+ breast cancer who developed bilateral leg weakness and loss of walking due to severe thoracic spinal cord compression. Imaging showed progressive epidural and intradural tumors, whereas osseous metastases remained stable. We performed single-cell RNA sequencing on tumor samples from both compartments after surgical decompression, analyzing cell types, immune subclustering, gene expression, and tumor module scores.

**Results:**

Sequencing revealed 12 distinct cell populations in both epidural and intradural metastases, including tumor epithelial, neural, glial, macrophages, myeloid, cytotoxic T, NK, and proliferating cells. While major cell types were similar, their composition and gene expression differed. Epidural cytotoxic T and NK cells expressed higher levels of GZMB, PRF1, NKG7, KLRD1, and GNLY. Macrophages split into 2 groups: a C1QA/C1QB-enriched subset mainly in the intradural compartment and an activated, inflammatory, antigen-presenting group enriched in the epidural compartment with IL1B, HLA-DRA, CD74, and other genes. Tumor epithelial cells also differed: intradural cells showed increased immune gene expression, antigen presentation, and interferon expression.

**Conclusion:**

The study shows cellular and transcriptional differences within a single spinal metastasis, influenced by local anatomy. These findings suggest that the microenvironment impacts immune and tumor states, guiding future research on metastasis and therapy responses.

Key Points

- Single-cell RNA sequencing identifies compartment-associated immune and tumor transcriptional heterogeneity within a contiguous spinal metastasis.
- Epidural and intradural metastases exhibit distinct immune compositions, tumor transcriptional programs, and module scores despite their close anatomical proximity.

Importance of the Study

This study demonstrates how the local spinal microenvironment is associated with immune and tumor transcriptional states within a single contiguous metastatic lesion. By performing single-cell RNA sequencing of matched epidural and intradural metastases from a patient with estrogen receptor (ER)+/progesterone receptor (PR+) metastatic breast cancer, we generated a comprehensive cellular atlas of both compartments, including tumor epithelial, immune, neural/glial, and proliferative populations. Immune reclustering identified compartment-associated differences in cytotoxic lymphocyte and macrophage populations, while tumor-specific differential expression analysis revealed distinct transcriptional programs and module scores between epidural and intradural tumor cells. Although these findings are derived from a single patient and require validation in larger cohorts, they provide new insight into how local anatomical context may influence the biology of spinal metastases. Collectively, this work highlights the importance of considering spatial context when interpreting the metastatic tumor microenvironment and provides a foundation for future mechanistic studies.

Spinal metastases are estimated to occur in up to 40% of cancer patients.^1^ However, there is rarely a distinction made between the anatomical compartments involved, which may include osseous disease, epidural extension causing spinal cord compression, and intradural tumor infiltration. These compartments differ substantially in their microenvironments, pathophysiology, and therapeutic challenges.^2^^,^^3^ The epidural space, rich in venous plexuses and adjacent to the vertebral body, is more commonly infiltrated by metastatic cells and may allow greater immune cell access.^4^^,^^5^ In contrast, the intradural space, surrounded by cerebrospinal fluid and neural tissue, is relatively immune privileged, which may contribute to decreased sensitivity to immunotherapy.^6^^,^^7^ Additionally, intradural metastases often present with direct involvement of the spinal cord or nerve roots, leading to more severe neurologic symptoms and complicating treatment strategies.^7^

Intradural metastases occur in approximately 5%-6% of spinal metastatic cases and remain particularly challenging to treat because of their location within the central nervous system.^8^ Surgical access is often limited, radiation efficacy is variable, and the role of systemic therapies remains incompletely understood.^8-10^ Consequently, the biological factors underlying their aggressive clinical behavior remain largely unknown.

Recent advances in single-cell RNA sequencing have enabled high-resolution characterization of the tumor microenvironment and have demonstrated substantial cellular heterogeneity both between and within metastatic lesions.^11^^,^^12^ Most studies, however, have compared tumors from different patients or anatomically distinct metastatic sites, making it difficult to distinguish true biological differences from interpatient variability. The rare occurrence of contiguous epidural and intradural disease within a single patient provides a unique opportunity to examine compartment-associated cellular states while minimizing interpatient heterogeneity.

Here, we performed single-cell RNA sequencing on matched epidural and intradural tumor specimens obtained from the same contiguous spinal metastasis. We first characterized the cellular composition of each compartment through unbiased clustering and canonical marker-based annotation, followed by immune-specific re-clustering to define lymphoid and myeloid populations. We then performed tumor-specific differential gene expression and module score analyses to identify transcriptional programs associated with each anatomical compartment. As this study represents a single heavily pretreated patient, our findings are intended to be hypothesis-generating and to provide a foundation for future investigations into the biological basis of compartment-specific behavior in spinal metastases.

## Methods

### Tissue Collection and Single-Cell RNA Sequencing

Fresh epidural and intradural tumor specimens were obtained under Memorial Sloan Kettering Cancer Center (MSKCC) Institutional Review Board-approved protocol 17-593, titled “Defining the Immunological Tumor Microenvironment on Metastatic and Primary Spine Tumors.” Clinical information was abstracted from medical records and de-identified. Informed consent was obtained from the patient. The epidural specimen consisted of tumor immediately external to the dura, whereas the intradural specimen was obtained following durotomy from grossly visible intradural tumor. Samples were immediately transported and mechanically dissociated with 5 mL of digestion buffer (2 mL Collagenase I [7.5 mg/mL], 1.49 mg DNase I, 8 mL 1% BSA in HBSS++) for 20 minutes at 37°C with rotation. After digestion, the material was strained, washed with 5 mL of SMEM + 2% FBS, and resuspended in 1 mL of SMEM + 2% FBS. Red blood cells were lysed with 10 mL of 1× Pharm Lysis for 1 minute, neutralized with 10 mL of SMEM + 2% FBS, and strained again. Samples were resuspended in 0.05% BSA in PBS and submitted for scRNA sequencing.

Single-cell libraries were generated using the Chromium Single Cell 3’ Gene Expression platform (10× Genomics, Pleasanton, CA) following the manufacturer’s protocol. Libraries were sequenced on an Illumina NovaSeq platform with a target depth of approximately 50,000 reads per cell.

### Single-Cell RNA Sequencing Processing

Raw sequencing data were processed using Cell Ranger (10× Genomics) and aligned to the human reference genome to generate gene-cell count matrices. Downstream analyses were performed using Seurat (v5.0) in R (v4.4.1).

Cells with fewer than 200 detected genes or greater than 10% mitochondrial transcript content were excluded from downstream analyses. Following quality control, normalized gene expression matrices from the epidural and intradural samples were integrated into a single Seurat object for downstream analyses. Data were log-normalized, the 2,000 most variable genes were identified, and principal component analysis (PCA) was performed using the variable features. Uniform Manifold Approximation and Projection (UMAP) was used for visualization, and unsupervised graph-based clustering was performed using the Louvain algorithm.

Quality-control metrics, including cell number, median detected genes, median unique molecular identifiers (UMIs), and mitochondrial transcript percentage for each sample, are provided in Table 1.

**Table 1.** Summary of single-cell RNA sequencing quality-control metrics

Sample	Cells passing QC	Median genes	Median UMIs	Median mito percent	Mean genes	Mean UMIs
Epidural	1076	994.5	1210.5	1.6	2329.2	10013.1
Intradural	1594	275	324	3.33	696.1	3237.5

Number of cells retained after quality-control filtering together with median and mean genes detected per cell, median and mean unique molecular identifier (UMI) counts, and median mitochondrial transcript percentage for the epidural and intradural specimens.

### Cell-Type Annotation

Major cellular populations were identified using canonical marker gene expression together with cluster-specific differentially expressed genes. Tumor epithelial cells were identified based on coordinated expression of epithelial and luminal breast cancer markers, including EPCAM, KRT8, KRT18, KRT19, MUC1, ESR1, PGR, and CD24. Neural and glial populations were identified using established neural lineage markers, including GFAP, AQP4, MBP, PLP1, SOX10, and RBFOX3. Macrophage/myeloid populations were identified by coordinated expression of canonical myeloid and macrophage markers, including LYZ, LST1, AIF1, CD68, FCGR3A, APOE, C1QA, C1QB, and C1QC, with inflammatory macrophage populations further characterized by expression of IL1B, OSM, PLAU, TREM1, HLA-DRA, CD74, SPP1, and GPNMB. Cytotoxic T/NK cells were identified by expression of lymphoid and cytotoxic effector genes, including PTPRC, CD3D, CD3E, TRAC, NKG7, GNLY, PRF1, GZMB, and KLRD1. Cycling/proliferative populations were identified by enrichment of cell cycle-associated genes, including MKI67, TOP2A, UBE2C, CDC20, KIF11, KIF20A, and KIF2C. Clusters lacking sufficient canonical marker expression for confident assignment were conservatively designated as unclassified.

### Immune Cell Subclustering

Clusters annotated as macrophage/myeloid or cytotoxic T/NK populations based on canonical lineage markers were subsetted and reanalyzed independently. Clusters corresponding to cytotoxic T/NK cells and macrophage/myeloid cells were subsetted and reanalyzed independently. Cell identities were confirmed using established lymphoid and myeloid marker genes, including PTPRC, CD3D, CD3E, TRAC, NKG7, PRF1, GNLY, LYZ, LST1, AIF1, FCGR3A, C1QA, C1QB, C1QC, and APOE. Macrophage populations were further characterized using inflammatory and antigen-presentation-associated genes, including IL1B, OSM, PLAU, TREM1, HLA-DRA, CD74, and APOE.

### Differential Gene Expression and Module Score Analysis

Tumor epithelial cells were analyzed separately to identify transcriptional differences between epidural and intradural compartments. Differential gene expression analysis was performed using Seurat’s FindMarkers function with the Wilcoxon rank-sum test implemented in Seurat. Genes with an adjusted *P*-value <.05 were considered significantly differentially expressed.

To characterize biological processes associated with each compartment, module scores were calculated using AddModuleScore in Seurat for predefined gene signatures representing antigen presentation, interferon response, ­epithelial/luminal differentiation, stress/hypoxia, and extracellular matrix remodeling. Module scores were compared between epidural and intradural tumor cells using two-sided Wilcoxon rank-sum tests.

### Module Score Gene Sets

Gene signatures were manually curated to represent biological processes relevant to metastatic breast cancer and tumor-immune interactions. The antigen presentation signature included genes involved in major histocompatibility complex (MHC) antigen processing and presentation (HLA-A, HLA-B, HLA-C, HLA-DRA, HLA-DRB1, CD74, B2M, TAP1, and TAP2). The interferon response signature comprised canonical interferon-stimulated genes (IFITM1, IFITM3, IFI6, IFI27, ISG15, MX1, OAS1, STAT1, and IRF7). Epithelial/luminal differentiation was assessed using established epithelial and luminal breast cancer markers (EPCAM, KRT8, KRT18, KRT19, MUC1, ESR1, PGR, and CD24). The stress/hypoxia signature included genes associated with cellular adaptation to hypoxic stress (HIF1A, VEGFA, CA9, LDHA, SLC2A1, BNIP3, and EGLN3), whereas the extracellular matrix remodeling signature consisted of genes involved in matrix organization and remodeling (COL1A1, COL1A2, COL3A1, FN1, SPARC, MMP2, MMP9, and TIMP1). Module scores were calculated using Seurat’s AddModuleScore function and were used to provide transcriptional interpretation of tumor-cell transcriptional differences identified by differential expression analysis rather than to classify individual cell types.

### Statistical Analysis

Comparisons between groups were performed using two-sided Wilcoxon rank-sum tests unless otherwise specified. *P*-values were adjusted for multiple hypothesis testing using the Benjamini-Hochberg false discovery rate correction where appropriate. Statistical significance was defined as an adjusted *P*-value <.05.

## Results

### Clinical Presentation and Tissue Acquisition

A 61-year-old female with a history of metastatic breast cancer (estrogen receptor [ER]+ and progesterone receptor [PR]+) presented with progressive bilateral lower extremity weakness and difficulty ambulating, requiring a wheelchair for several weeks. She had known widespread metastatic disease, including the lungs, lymph nodes, bones, and brain. She had previously undergone 3 separate courses of radiation therapy for symptomatic spinal metastases, including the cervical and thoracic spine, as well as kyphoplasty treatment at T7 and T9 for symptomatic pathologic fractures.

**Figure 1.**
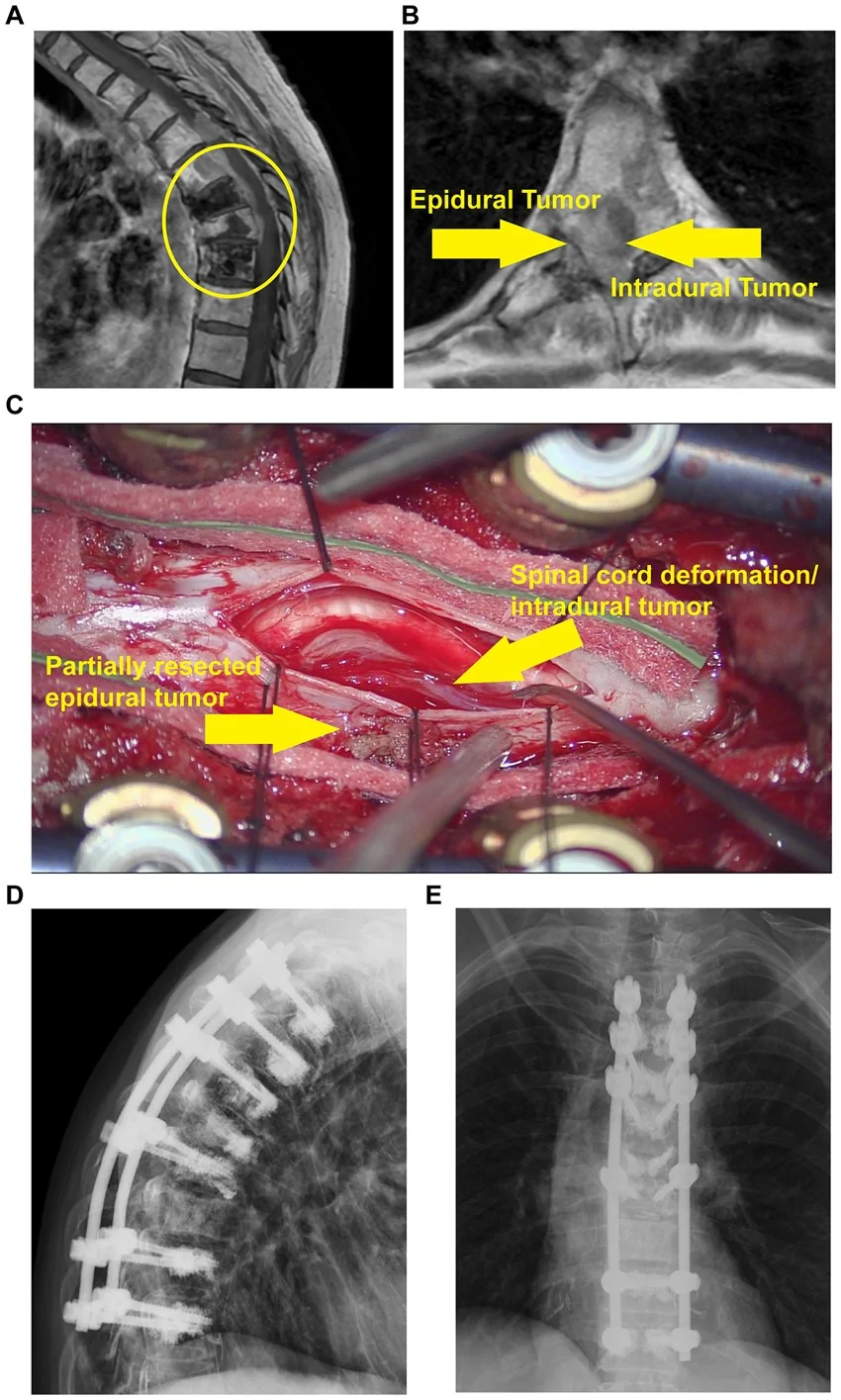
Clinical presentation of ER^+^/PR^+^ metastatic breast cancer to the spine. (A) Sagittal MRI with gadolinium contrast enhancement demonstrating the multilevel disease with multilevel fractures and circumferential epidural spinal cord compression. (B) Axial MRI with gadolinium contrast enhancement demonstrating intradural and epidural disease resulting in severe spinal cord compression. (C) Intraoperative snapshot demonstrating residual epidural disease as well as intradural disease compressing the deformed spinal cord. (D) Lateral and (E) Anterior-posterior x-rays showing the postoperative hardware with cement augmentation.

An MRI was performed, demonstrating progressive epidural disease causing severe spinal cord compression and early signs of spinal cord injury at T5-T8 with a combination of increased epidural disease as well as suspicion of intradural tumor extension (Figure 1A and B). Given her progressive neurological decline, following a multidisciplinary tumor board discussion, the recommendation was for surgical decompression for neurologic salvage and pain palliation, followed by salvage stereotactic re-irradiation in hopes of more durable local tumor control.

She underwent T4-T9 posterolateral spinal cord decompression, which included multilevel laminectomies, bilateral facetectomies, resection of epidural tumor, T4-5 microscopic resection of intradural metastases (ie leptomeningeal disease), T4-T11 posterior instrumented fusion with cement-augmented pedicle screws and connecting rods, as well as paraspinal muscle advancement flaps (Figure 1C-E).

Surgery was noteworthy because of the intradural tumor extension, which is very uncommon in metastatic spine disease, as epidural tumors rarely penetrate the dura in such cases. This intradural extension appeared likely to have occurred through perineural infiltration of the nerve root sleeve (Figure 1C).

The patient underwent a decompressive laminectomy with resection of both epidural and intradural tumor, allowing independent collection of viable tissue from each anatomical compartment for single-cell RNA sequencing (Figure 1E).

Following surgery, the patient regained ambulation with the assistance of a rolling walker and maintained bowel and bladder control. She underwent postoperative salvage stereotactic radiation in 5 fractions and remained ambulatory with no further spinal disease progression until she succumbed to her disease 9 months later.

Intraoperative specimens from four separate osseous sites with macroscopic abnormalities showed no viable tumor on histopathologic examination, likely representing previously irradiated, necrotic bone. However, both epidural and intradural soft tissue specimens demonstrated viable metastatic carcinoma with morphology and immunohistochemical profile (ER+, PR+) consistent with the known breast primary.

### Single-Cell Transcriptomic Profiling Identifies Distinct Cellular Populations Within Contiguous Epidural and Intradural Metastases

Following quality control, 1,076 epidural cells and 1,594 intradural cells were retained for downstream analysis (Table 1). Unsupervised clustering identified 12 transcriptionally distinct clusters that were visualized using UMAP.

**Figure 2.**
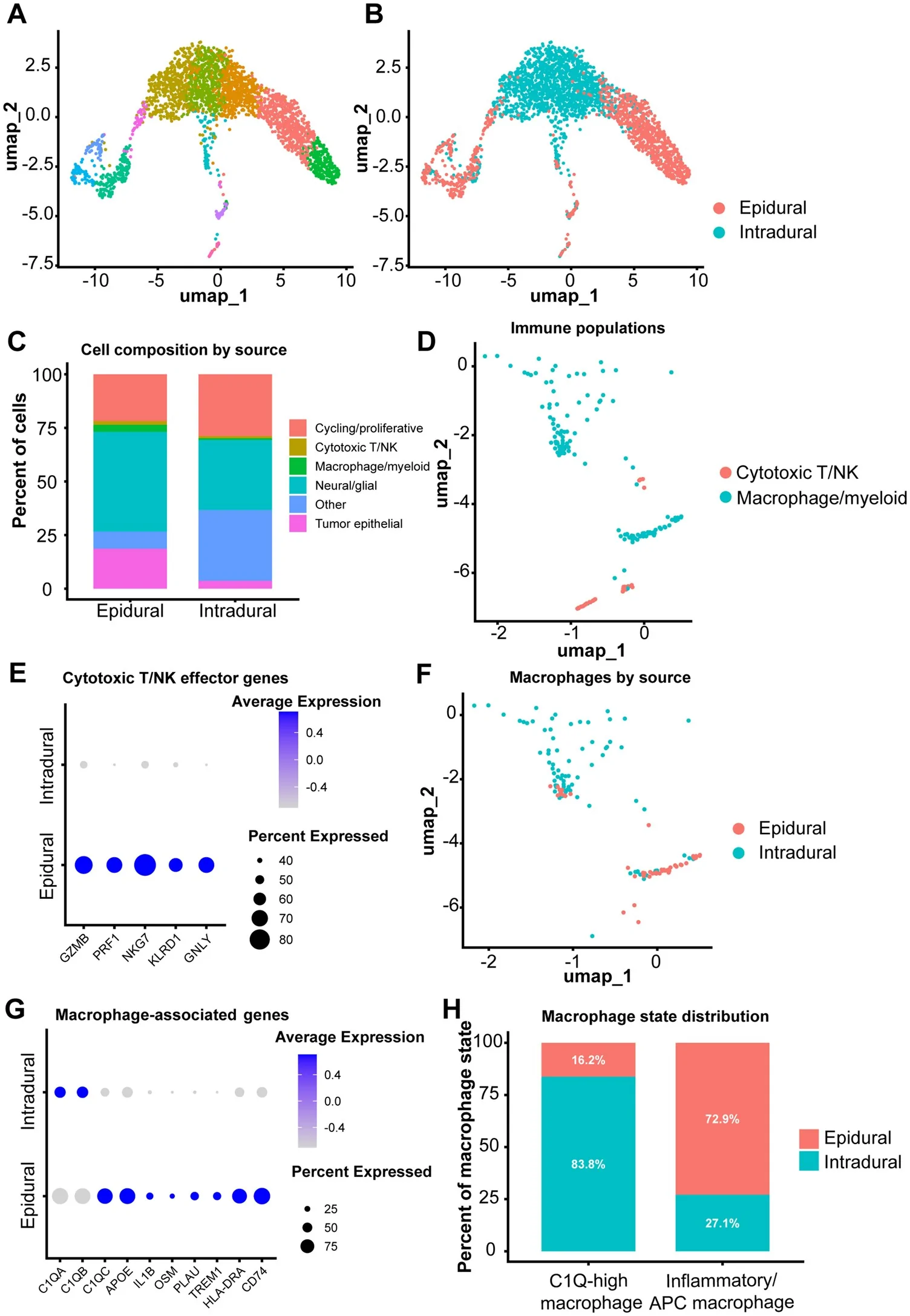
Single-cell transcriptomic analysis identifies compartment-associated immune cell populations within contiguous epidural and intradural spinal metastases. (A) UMAP visualization of all cells colored by transcriptional cluster following integration of epidural and intradural specimens. (B) UMAP colored by anatomical source demonstrating contributions of both compartments across multiple transcriptional clusters. (C) Relative cellular composition of epidural and intradural specimens by annotated major cell type. (D) UMAP of immune cells following immune-specific reclustering identifying cytotoxic T/NK and macrophage/myeloid populations. (E) Dot plot demonstrating increased expression of canonical cytotoxic effector genes (GZMB, PRF1, NKG7, KLRD1, and GNLY) in epidural cytotoxic T/NK cells relative to intradural cytotoxic T/NK cells. Dot size indicates the percentage of expressing cells and color intensity indicates scaled average expression. (F) UMAP of macrophage/myeloid cells colored by anatomical source. (G) Dot plot comparing the expression of representative macrophage-associated genes between epidural and intradural macrophage populations, including a C1QA/C1QB-enriched macrophage population and an activated inflammatory/antigen-presenting macrophage population expressing IL1B, OSM, PLAU, TREM1, HLA-DRA, CD74, and APOE. (H) Relative distribution of macrophage transcriptional states across anatomical compartments, demonstrating enrichment of the C1Q-high macrophage population within the intradural specimen and enrichment of the inflammatory/antigen-presenting macrophage population within the epidural specimen.

To facilitate biological interpretation, clusters were annotated using canonical lineage markers alongside cluster-specific differentially expressed genes (Figure 2A and Supplementary Figure S1A). The principal cellular populations included neural/glial cells, tumor epithelial cells, macrophage/myeloid cells, cytotoxic T/NK cells, cycling/proliferative cells, and a minor unclassified subset lacking sufficient expression of canonical markers for definitive categorization (Supplementary Figure S1B). Tumor epithelial populations were characterized by the expression of EPCAM, KRT8, KRT18, KRT19, MUC1, ESR1, and PGR. In contrast, macrophage/myeloid populations expressed LYZ, LST1, AIF1, FCGR3A, CD68, C1QA, C1QB, and C1QC. Cytotoxic lymphocytes exhibited expressions of CD3D, CD3E, TRAC, NKG7, GNLY, PRF1, GZMB, and KLRD1 (Supplementary Figure S1C and D).

Visualization of the 2 specimens independently demonstrated that both epidural and intradural samples contributed to multiple transcriptional clusters, albeit with differences in their relative cellular composition (Figure 2B). Quantification of cell-type frequencies revealed compartment-associated variations in the abundance of immune and tumor cell populations, notwithstanding their close anatomical proximity (Figure 2C).

### Immune Subclustering Reveals Compartment-Associated Lymphoid and Myeloid Transcriptional Programs

To further characterize the immune microenvironment, immune cells were subset and reclustered independently (Figure 2D). Immune reclustering identified macrophage/myeloid and cytotoxic T/NK populations, which were annotated using canonical lineage markers.

Epidural cytotoxic T/NK cells exhibited an increased expression of canonical cytotoxic effector genes, including GZMB, PRF1, NKG7, GNLY, and KLRD1, compared to their intradural counterparts (Figure 2E). Immune reclustering further distinguished 2 transcriptionally distinct macrophage populations (Figure 2F). One population, enriched for C1QA/C1QB, was predominantly observed in the intradural specimen, whereas a second population, characterized by the expression of genes involved in inflammation and antigen presentation—such as IL1B, OSM, PLAU, TREM1, HLA-DRA, CD74, and APOE—was more abundant in the epidural compartment (Figure 2G). These findings imply that the epidural and intradural compartments exhibit distinct macrophage transcriptional states, as well as differences in cytotoxic lymphocyte-associated programs.

Given that this analysis reflects the transcriptional profiling of a single patient, these observations should be regarded as indicative of compartment-associated immune states rather than conclusive functional phenotypes.

### Tumor Epithelial Cells Demonstrate Compartment-Associated Transcriptional Differences

**Figure 3.**
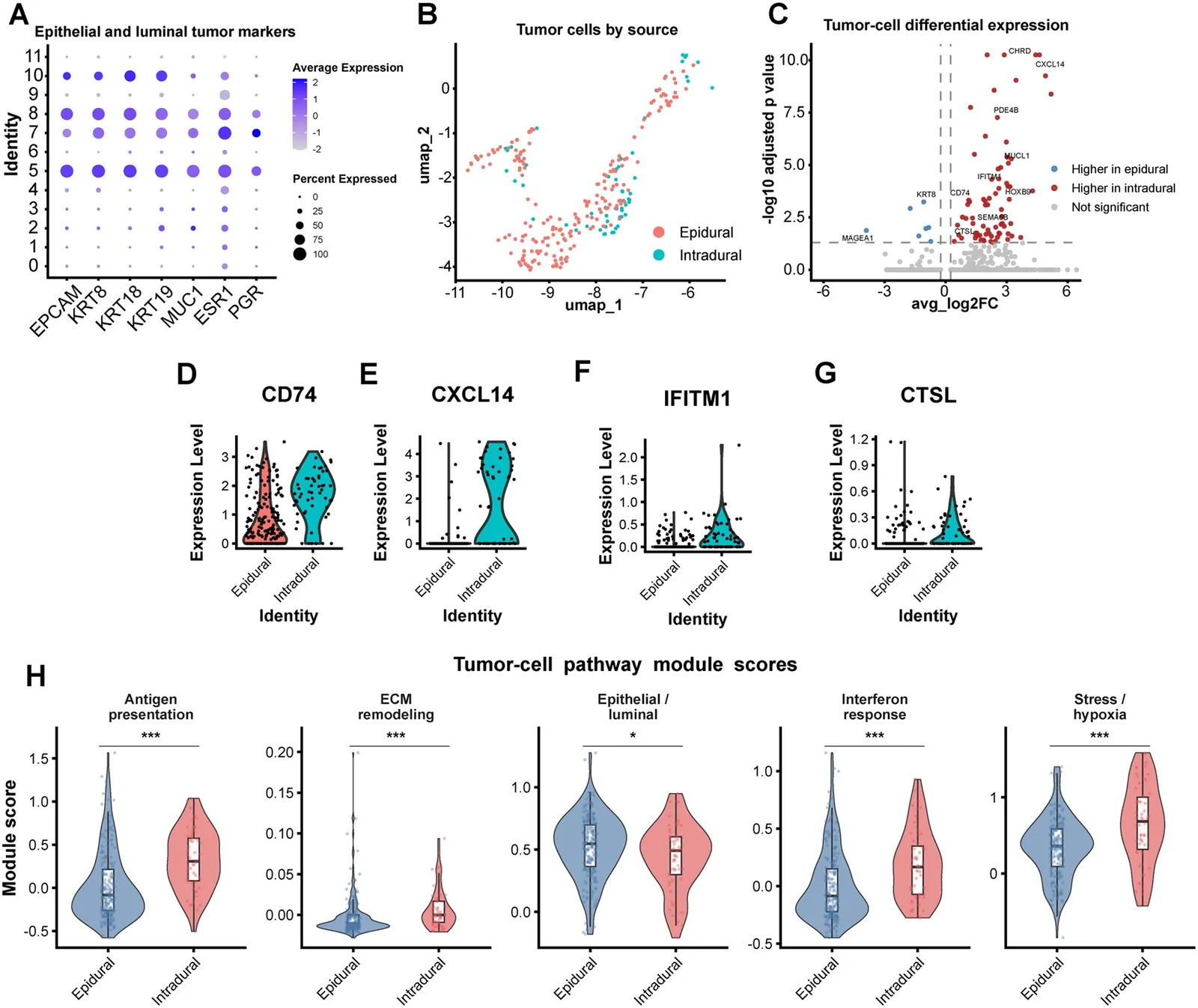
Tumor epithelial cells demonstrate compartment-associated transcriptional differences. (A) Dot plot demonstrating expression of canonical epithelial and luminal breast cancer markers (EPCAM, KRT8, KRT18, KRT19, MUC1, ESR1, and PGR) across tumor-associated clusters, supporting tumor cell annotation. Dot size indicates the percentage of expressing cells and color intensity indicates scaled average expression. (B) UMAP of tumor epithelial cells colored by anatomical source. (C) Volcano plot showing differentially expressed genes between epidural and intradural tumor epithelial cells. Genes shown in red are significantly enriched in intradural tumor cells, whereas genes shown in blue are enriched in epidural tumor cells. (D-G) Violin plots illustrating representative differentially expressed genes identified between compartments, including CD74, CXCL14, IFITM1, and CTSL. (H) Module score analysis demonstrating compartment-associated pathway activity within tumor epithelial cells. Intradural tumor cells exhibited increased antigen-presentation and interferon-response module scores, whereas epidural tumor cells demonstrated relatively greater epithelial/luminal differentiation. Additional differences were observed in extracellular matrix remodeling and stress/hypoxia-associated transcriptional programs. Statistical comparisons were performed using two-sided Wilcoxon rank-sum tests.

To determine whether tumor cells varied across different anatomical compartments, tumor epithelial cells were analyzed separately and identified through canonical epithelial and luminal gene markers (Figure 3A and B). Differential expression analysis revealed distinct transcriptional profiles between epidural and intradural tumor cells (Figure 3C).

Intradural tumor cells exhibited heightened expression of genes associated with antigen presentation and immune interaction, notably including CD74, as well as elevated levels of CXCL14, CTSL, and IFITM1, underscoring transcriptional divergence between the two compartments despite their anatomical continuity (Figure 3C-G).

Module score analysis further substantiated the enrichment of compartment-associated transcriptional programs within tumor cells (Figure 3H). Intradural tumor cells showed elevated scores in antigen-presentation and interferon-response modules, whereas epidural tumor cells exhibited comparatively higher enrichment of epithelial/luminal differentiation programs. Additionally, signatures associated with stress/hypoxia and extracellular matrix remodeling varied across compartments, indicating that the local anatomical context correlates with distinct transcriptional states of the tumor.

Collectively, these findings demonstrate that both immune and tumor compartments exhibit transcriptional heterogeneity across contiguous epidural and intradural metastases within the same patient.

### Integrated Analysis Identifies Compartment-Associated Cellular Ecosystems

Integration of immune and tumor analyses revealed that the epidural and intradural compartments are characterized by distinct cellular ecosystems, despite their close anatomical proximity. The epidural compartment was enriched for cytotoxic T/NK cell-associated transcriptional programs and an activated inflammatory/antigen-presenting macrophage population expressing IL1B, HLA-DRA, CD74, APOE, PLAU, OSM, and TREM1. Conversely, the intradural compartment was enriched for a macrophage population marked by C1QA/C1QB and for tumor cells exhibiting heightened transcriptional activity related to antigen presentation and interferon response. These findings collectively indicate that both the immune and malignant compartments experience compartment-specific transcriptional adaptations within contiguous spinal metastases.

## Discussion

The microenvironment of spinal metastases is anatomically diverse. Nevertheless, epidural and intradural metastases are often grouped together, despite significant differences in their biology, response to treatment, and clinical behavior. In this study, we used the uncommon occurrence of contiguous epidural and intradural metastatic disease in the same patient to analyze cellular and transcriptional heterogeneity, thereby reducing the confounding effects of interpatient variability. By employing comprehensive methods including unbiased single-cell clustering, canonical cell-type annotation, immune subclustering, tumor-specific differential expression analysis, and transcriptional interrogation, we identified differences associated with these compartments in both immune and tumor cell populations, despite their close anatomical proximity.

Our analysis demonstrated that the epidural compartment was enriched for cytotoxic lymphocyte-associated transcriptional programs alongside an activated inflammatory/antigen-presenting macrophage population, whereas the intradural compartment was enriched for a transcriptionally distinct C1QA/C1QB-enriched macrophage population. Independent analysis of tumor epithelial cells further revealed compartment-associated transcriptional divergence, including differential expression of genes involved in antigen presentation and epithelial differentiation, together with enrichment of antigen-presentation and interferon-response transcriptional programs. Collectively, these findings suggest that the local anatomical context is associated with a coordinated transcriptional adaptation of both immune and tumor cell populations within a single contiguous metastatic lesion.

The observation of increased cytotoxic lymphocyte-associated transcriptional programs within the epidural compartment is consistent with the greater vascularity and immune accessibility characteristic of the epidural space. Further immune reclustering identified 2 transcriptionally distinct macrophage populations exhibiting differential anatomical enrichment, including a C1QA/C1QB-enriched macrophage population that predominantly occupied the intradural specimen and an activated inflammatory/antigen-presenting macrophage population that was enriched in the epidural compartment. Alongside the compartment-associated transcriptional differences observed in tumor epithelial cells, these findings suggest that both immune and malignant cell populations undergo local transcriptional adaptation across contiguous spinal compartments. These differences were identified through differential expression analyses performed within individually annotated tumor and immune cell populations, rather than through comparisons of bulk tissue composition. Although neural and glial cell populations were identified in the integrated dataset, they were not the focus of downstream differential expression analyses. Since both specimens were obtained from contiguous regions of the same lesion, these differences are less likely to reflect interpatient or intertumoral heterogeneity; however, they do not exclude the possibility that anatomical factors or variations in disease progression or prior treatment may influence these observations.

Furthermore, tumor-specific differential expression analysis revealed that malignant cells also exhibited transcriptional differences across compartments. The elevated expression of genes such as CD74, CXCL14, IFITM1, and other immune-related genes within intradural tumor cells, combined with the enrichment of antigen-presenting and interferon-response pathways, suggests that tumor cells may undergo compartment-specific transcriptional adaptation in conjunction with alterations in the surrounding immune microenvironment. Determining whether these changes are a response to immune pressure, local stromal interactions, or treatment-induced adaptations will necessitate further research.

Our findings should be interpreted within the context of several significant limitations. Firstly, this study represents a single patient with heavily pretreated metastatic breast cancer, which includes multiple prior courses of radiation therapy. These interventions may have affected both immune composition and the tumor’s transcriptional state. Therefore, the compartment-associated differences identified herein should not be regarded as inherent characteristics of the epidural or intradural microenvironments but rather as observations linked to this specific clinical setting. Future research involving larger patient cohorts and treatment-naïve specimens will be necessary to determine the extent to which these findings are applicable to a broader population with spinal metastases.

Secondly, single-cell RNA sequencing assesses the transcriptional state rather than functional cellular activity. Although cytotoxic lymphocytes exhibit elevated expressions of canonical effector genes such as PRF1, GZMB, and GNLY, these data do not confirm functional cytolytic activity or direct interactions with tumor cells. Complementary methodologies, including multiplex immunofluorescence, spatial transcriptomics, or spatial proteomic analyses, are necessary to ascertain whether these transcriptional programs are indicative of functional immune responses within the tissue microenvironment.

Third, enzymatic dissociation and single-cell library preparation may preferentially recover certain cell populations while underrepresenting fragile cell types or inducing stress-associated transcriptional responses. Although identical processing workflows were applied to both specimens, technical factors cannot be entirely ruled out as contributors to the observed differences.

Finally, the intradural component may represent a later stage of local disease evolution rather than solely a distinct anatomical niche. Consequently, some of the transcriptional differences observed between epidural and intradural compartments may reflect temporal progression in addition to local microenvironmental influences. Longitudinal sampling and larger cohort studies will be required to distinguish spatial from temporal determinants of metastatic evolution within the spine.

Despite these limitations, this case provides a unique opportunity to examine cellular heterogeneity across 2 contiguous metastatic compartments within the same patient. Such specimens are rarely available, particularly from matched epidural and intradural disease collected simultaneously during surgery. The ability to compare these anatomically distinct but genetically related lesions minimizes many sources of biological variability that complicate comparisons across patients or metastatic sites and provides a valuable framework for investigating how local tissue context shapes metastatic behavior.

In conclusion, single-cell transcriptomic profiling of matched epidural and intradural spinal metastases identified compartment-associated differences in immune composition and tumor transcriptional programs within a single contiguous lesion. Although these observations require validation in larger cohorts and with complementary spatial and functional approaches, they provide new insight into the complexity of the spinal metastatic microenvironment and generate hypotheses regarding how local anatomical context may influence metastatic progression and therapeutic response.

## Supplementary Material

vdag225_Supplementary_Data

## Data Availability

The single-cell RNA sequencing data generated during this study will be made publicly available in the Gene Expression Omnibus (GEO) upon publication. Additional data supporting the findings of this study are available from the corresponding author upon request.
